# Double-Lung Transplant With Delayed Nuss Bar Prostheses for Pectus Excavatum Deformity

**DOI:** 10.1016/j.atssr.2025.06.005

**Published:** 2025-06-25

**Authors:** Amer Alzahrani, Mario Castro-Medina, Masashi Furukawa, Pablo G. Sanchez

**Affiliations:** 1 Division of Cardiothoracic Transplantation, Department of Cardiothoracic Surgery, University of Pittsburgh, Pittsburgh, Pennsylvania; 2 Division of Pediatric Cardiothoracic Surgery, Department of Cardiothoracic Surgery, University of Pittsburgh, Pittsburgh, Pennsylvania; 3 Section of Thoracic Surgery, Department of Surgery, The University of Chicago Biological Sciences Division, Chicago, Illinois

## Abstract

Severe pectus excavatum and prior talc pleurodesis are traditionally considered relative contraindications to lung transplantation. A 36-year-old woman with idiopathic pulmonary fibrosis, pectus excavatum with a Haller Index of 6.7, and prior bilateral talc pleurodesis underwent successful bilateral lung transplantation. Chest closure was delayed due to right heart compression. On postoperative day 6, staged chest wall reconstruction with 2 Nuss bars was performed. She recovered without complications, was discharged on day 70, and remains off oxygen 910 days after transplant. With multidisciplinary planning and staged reconstruction, lung transplantation is feasible in select patients with complex chest wall pathology.

Severe chest wall deformities and prior talc pleurodesis are considered potential contraindications to lung transplantation due to technical complexity.^1^ Challenges include difficult lung explantation, limited intrathoracic space, reduced chest wall compliance, and cardiopulmonary instability. Despite these concerns, reports suggest that with meticulous planning and multidisciplinary surgical strategies, transplantation may be feasible in selected cases.^2^, ^3^, ^4^

We describe a 36-year-old woman with idiopathic pulmonary fibrosis (IPF), severe pectus excavatum, and 6 prior thoracoscopic talc pleurodesis procedures who underwent successful bilateral lung transplantation. A staged chest closure with delayed chest wall reconstruction using the Nuss procedure was used. We present intraoperative and postoperative data, address prosthetic use and infection concerns, and explain the rationale for delayed repair.

A 36-year-old woman with IPF (diagnosed in 2010) and chronic respiratory failure was evaluated for lung transplantation. She had recurrent spontaneous pneumothoraces from age 14, treated with 6 bilateral thoracoscopic talc pleurodesis procedures, the last performed 10 years prior. Her condition worsened after pregnancy. At evaluation, she required 15 L/min of oxygen with exertion and 7 L/min at rest. A 6-minute walk test was aborted due to hypoxemia. Pulmonary function testing showed a restrictive defect: forced vital capacity of 1.21 L (31% predicted) and forced expiratory volume in 1 second of 1.16 L (37% predicted). High-resolution computed tomography revealed bilateral fibrosis and severe pectus excavatum with a Haller Index of 6.7 (Figure 1; Supplemental Video 1). Her family history was significant for pulmonary fibrosis: her mother received a bilateral lung transplant in 2020, and her father and brother both had spontaneous pneumothoraces.

**Figure 1:**
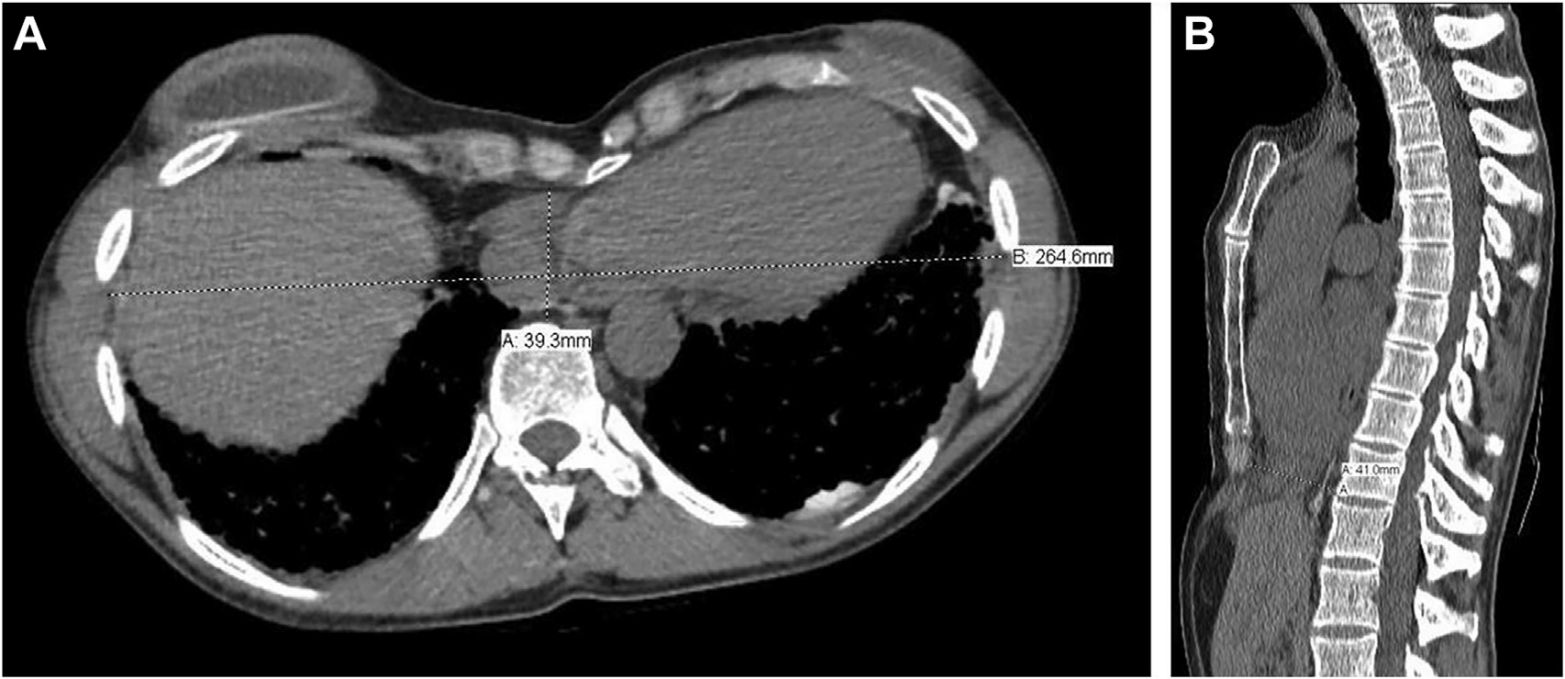
Preoperative computed tomographic scan demonstrates severe pectus excavatum with a Haller Index of 6.7. (A) Axial view. (B) Sagittal view.

A multidisciplinary team planned bilateral lung transplantation with delayed pectus repair using the Nuss procedure. Donor-recipient height match was favorable (recipient, 165 cm; donor, 157 cm female), and size reduction was not needed. The transplant was performed through a clamshell incision with central cardiopulmonary bypass. Planned bilateral thoracotomies were abandoned due to leftward mediastinal displacement. Bilateral saline breast implants were removed. Explantation was challenging due to dense adhesions.

The patient received 9 units of packed red blood cells, 6 units of fresh frozen plasma, 4 units of platelets, 4 units of cryoprecipitate, and 4 liters of crystalloid. Bypass time was 299 minutes. Ischemic times were left, 348 minutes (warm 61 minutes); right, 473 minutes (warm 48 minutes). Owing to surgical complexity and anticipated chest wall expansion, delayed chest closure was planned.

On postoperative day (POD) 6, chest wall reconstruction was performed in the operating room. A 7-inch and 8-inch Nuss bar were placed subpectorally and subcutaneously, curved using a bar bender, and secured with size 5 TI-CRON (Medtronic) sutures. Closure was completed with a FiberWire System (Arthrex). Intraoperative transesophageal echocardiography confirmed preserved right ventricular function.

Postoperative recovery included elective tracheostomy on POD 9, weaned off mechanical ventilation POD 17, intensive care unit discharge on POD 19, and decannulation on POD 44. She was discharged home on POD 70. Follow-up computed tomography showed correct bar positioning and Haller Index improvement to 2.9 (Figure 2; Supplemental Video 2). The bars were removed electively on POD 659. A follow-up chest radiograph (Figure 3; Supplemental Video 3) demonstrated a normalized thoracic contour. As of POD 910, she remains clinically stable and off oxygen. Histopathology of the explanted lungs revealed fibrosing interstitial pneumonia, bronchiolectasis, interstitial emphysema, and fibrotic pleura with polarizable talc.

**Figure 2:**
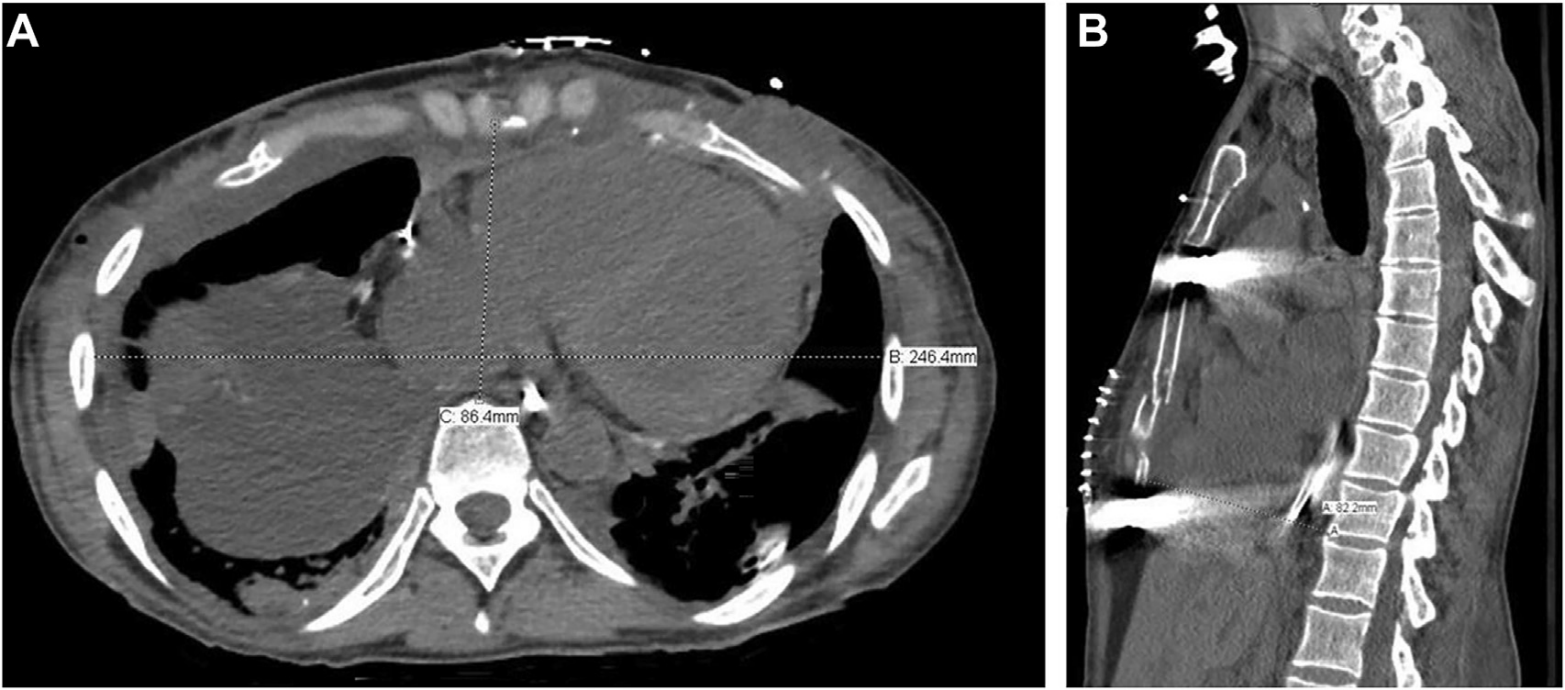
Postoperative computed tomographic scan after bilateral lung transplantation and Nuss bar placement shows improved thoracic configuration. (A) Axial view. (B) Sagittal view.

**Figure 3:**
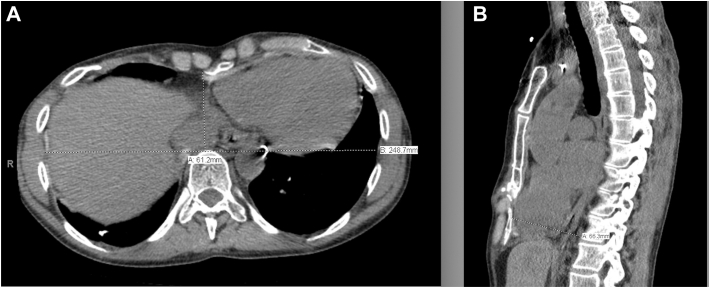
Follow-up computed tomographic scan after elective Nuss bar removal, demonstrates normalized chest wall architecture and sustained correction. (A) Axial view. (B) Sagittal view.

The patient expressed deep gratitude to the surgical team. Although the early postoperative course was demanding, she is now fully active without supplemental oxygen. She credits the staged chest repair with supporting her long-term recovery.

## Comment

Severe pectus excavatum and prior talc pleurodesis present technical and physiological challenges in lung transplantation. These include limited thoracic space, distorted mediastinal structures, dense adhesions, and compromised chest wall compliance.^2^, ^3^, ^4^, ^5^ Despite these challenges, this case suggests that with thorough preoperative planning and multidisciplinary coordination, such barriers may be managed effectively. The presence of extensive adhesions significantly increased the complexity of the bilateral pneumonectomies; however, careful dissection under cardiopulmonary bypass facilitated safe explantation.

Delayed chest closure with staged Nuss bar placement allowed for safe reconstruction without hemodynamic compromise.^2^ Sternal elevation reduced right heart compression, confirmed by intraoperative echocardiography. This case supports the feasibility of the Nuss procedure in adult lung transplant recipients. Haller Index improvement (6.7 to 2.9) indicates effective remodeling, with stable bar position and uneventful removal suggesting durable outcomes. This case highlights the role of thorough preoperative assessment and multidisciplinary planning in anatomically complex lung transplants.

Although chest wall deformities and prior pleurodesis increase surgical complexity, successful transplantation may still be feasible in selected patients.^6^ However, given the high-risk nature of such cases, they should be undertaken only in high-volume centers with the full spectrum of surgical, critical care, and rehabilitation expertise required for successful management.
